# Influence of Mineral Oil-Based Nanofluids on the Temperature Distribution and Generated Heat Energy Inside Minimum Oil Circuit Breaker in Making Process

**DOI:** 10.3390/nano13131951

**Published:** 2023-06-27

**Authors:** Hesham S. Karaman, Adel Z. El Dein, Diaa-Eldin A. Mansour, Matti Lehtonen, Mohamed M. F. Darwish

**Affiliations:** 1 Department of Electrical Engineering, Faculty of Engineering at Shoubra, Benha University, Cairo 11629, Egypt; hesham.said@feng.bu.edu.eg; 2 Department of Electrical Engineering, Faculty of Energy Engineering, Aswan University, Aswan 81528, Egypt; azeinm2001@hotmail.com; 3 Faculty of Technological Industry and Energy, Thebes Technological University, Luxor 85863, Egypt; 4 Department of Electrical Power Engineering, Egypt-Japan University of Science and Technology (E-JUST), New Borg El-Arab City 21934, Alexandria, Egypt; 5 Department of Electrical Power and Machines Engineering, Faculty of Engineering, Tanta University, Tanta 31511, Egypt; 6 Department of Electrical Engineering and Automation, School of Electrical Engineering, Aalto University, 02150 Espoo, Finland

**Keywords:** insulating oil, nanofluids, minimum oil circuit breaker, thermal properties

## Abstract

The enhancement of the thermal properties of insulating oils has positively reflected on the performance of the electrical equipment that contains these oils. Nanomaterial science plays an influential role in enhancing the different properties of liquids, especially insulating oils. Although a minimum oil circuit breaker (MOCB) is one of the oldest circuit breakers in the electrical network, improving the insulating oil properties develops its performance to overcome some of its troubles. In this paper, 66 kV MOCB is modeled by COMSOL Multiphysics software. The internal temperature and the internally generated heat energy inside the MOCB during the making process of its contacts are simulated at different positions of the movable contact. This simulation is introduced for different modified insulating oils (mineral oil and synthetic ester oil) with different types of nanoparticles at different concentrations (0.0, 0.0025, 0.005, and 0.01 wt%). From the obtained results, it is noticed that the thermal stress on the MOCB can be reduced by the use of high thermal conductivity insulating oils. Nano/insulating oils decrease internal temperature and generate heat energy inside the MOCB by about 17.5%. The corresponding physical mechanisms are clarified considering the thermophoresis effect.

## 1. Introduction

**Figure 1:**
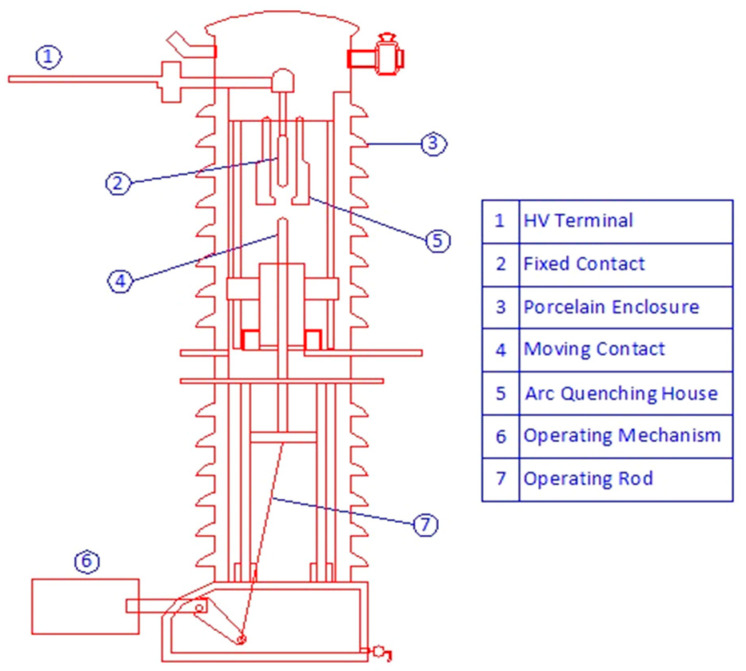
Internal view for minimum oil circuit breaker.

The minimum oil circuit breaker (MOCB) is one of the oldest circuit breakers. In this type of circuit breaker, the fixed and movable contacts are immersed in insulating oil. This oil has been used as an insulating, cooling, and arc-quenching medium. Figure 1 presents an internal view of one pole of the minimum oil circuit breaker. Due to the thermal and dielectric deterioration of this oil, alternative costly circuit breakers are used, such as sulfur hexafluoride (SF_6_) and vacuum circuit breakers, instead of oil circuit breakers that require repeated maintenance processes. If the thermal and dielectric properties of the oil used in MOCB are improved, its maintenance requirements will be less, and its lifetime will be longer, postponing its replacement with a new one. Regarding thermal properties, due to the high-temperature arc produced between MOCB contacts, the enhancement of the insulating oil thermal conductivity positively affects the performance of the MOCB by mitigating the thermal stress on its components. A high-temperature arc is produced due to the opening or closing of the circuit breaker contacts regardless of the type of arc quenching medium. Yangze et al. analyzed the arc spectrum between the contacts of the SF_6_ circuit breaker and presented that the temperature at the arc’s center reached 25,000 K [1]. In [2], the temperature profile for the produced arc due to the switching-off of the SF_6_ circuit breaker ranged between 10,000 K and 16,000 K. Jazini et al. studied the thermal performance of the contacts in circuit breakers and presented that the maximum temperature along the arc between the circuit breaker contacts is 40,273 K [3]. This high temperature causes an erosion of the contacts and shortens the lifetime of the circuit breaker as a whole.

Researchers recently studied the development of nanoparticle dispersion into the base insulating oil, known as nanofluids, to improve the dielectric and thermal properties of base oil [4,5]. For dielectric properties, using nanofluids enhanced breakdown strength [5,6], dielectric dissipation factor [5,7], partial discharge resistance [8], and creepage discharge [9]. For thermal properties, both the thermal conductivity and the heat transfer coefficient have been investigated. In [10], thermal conductivity improved by about 20% and 25% when a 0.05% volume fraction of multi-walled carbon nanotubes and spherical diamond nanoparticles were dispersed into the base oil. Moreover, multi-walled carbon nanotubes could improve the convective heat transfer coefficient under natural airflow up to about 26% when the input power was set at 120 W and up to 24% when the input power was set at 50 W [11]. In the same research, the convective heat transfer coefficient was improved under forced air flows up to about 17% and 25% for an input power of 120 W and 50 W, respectively. Although carbon nanotubes and diamond nanoparticles proved their effectiveness regarding thermal properties, they decreased the dielectric strength of the prepared nanofluids [10,11] or even caused a weak enhancement [12]. As a substitution, nanostructures of graphene or graphene oxide were used due to their superiority in enhancing both the thermal and dielectric properties of the prepared nanofluids. In [13,14], graphene oxide could improve the dielectric strength of the prepared nanofluids without monitoring thermal properties, while, in [15], graphene oxide could improve both the dielectric strength and thermal conductivity of prepared nanofluids. In [16,17], simultaneous enhancements in thermal conductivity and dielectric strength were obtained using graphene with insulating oil. In [16], graphene was used with transformer oil, exhibiting a maximum enhancement in thermal conductivity of about 30% at 0.01% weight fraction (0.01 wt%) and 328 K. At the same weight fraction, an enhancement in the dielectric strength of about 17% was achieved. In [17], using graphene with cottonseed oil could enhance the thermal conductivity by up to 18% at 0.01 wt% and 338 K and enhance the dielectric strength by up to 41% at the same weight fraction. The longer sonication time resulted in higher thermal conductivity due to possibly breaking up graphene nanosheet clusters. Saman et al. reported that conventional mineral oil can be electrically improved by the dispersing of plasma-treated alumina nanoparticles inside it. Where the increase in alumina wt% up to 0.1% achieved an increase in breakdown strength to 58 kV, nevertheless, the 0.3% weight percentage decreased the breakdown strength to 53 kV due to alumina nanoparticle agglomeration. Regarding thermal conductivity, the weight percentage of 0.1% of alumina has higher thermal conductivity than pure oil due to enhanced heat transferability [18]. In addition, Wanatasanappan et al. [19] investigated the thermophysical properties (thermal conductivity, density, and dynamic viscosity) of soybean oil, coconut oil, and palm oil-based hybrid nanofluids bonded with Al_2_O_3_-TiO_2_ nanoparticles. The results denote that palm oil mixed with nanofluids has superior thermophysical assets compared with the other two types, with the highest thermal conductivity and lowest density and viscosity. Moreover, Muzafar et al. [20] presented a study using three different nanomaterials (silica, alumina, titania) with different concentrations inside transformer oil. The presented results prove that alumina (Al_2_O_3_) has a higher breakdown strength of 32% at a concentration of 0.06 as compared to other samples. Furthermore, the loading of alumina with wt% from 0.5% to 3% enhances thermal conductivity at 35 °C from 0.135 W/m°C to 0.15 W/m°C, respectively.

Other types of nanoparticles could improve both thermal and dielectric properties of transformer oil-based nanofluids, such as calcium copper titanium oxide (CaCu_3_Ti_4_O_12_) [21] and barium titanate/TiO_2_ [22]. Regarding CaCu_3_Ti_4_O_12_ nanoparticles [21], they could enhance the thermal conductivity and AC breakdown strength of synthetic ester oil by about 10% and 42%. On the other hand, using barium titanate/TiO_2_ nanoparticles [22] enhanced the heat transfer coefficient of prepared nanofluids by about 33% and their dielectric strength by about 43%. Fernández et al. concluded that the loading of TiO_2_ and zinc oxide (ZnO) nanoparticles into insulating vegetable ester oil with wt.% concentration up to 0.16% achieved an enhancement of breakdown voltage of about 36% and 28% [23]. Furthermore, in [24], the authors reviewed the dispersion of aluminum oxide (Al_2_O_3_) nanoparticles into insulating oil with different concentrations, which causes thermal conductivity enhancement that is proportional to the concentration and temperature of nanoparticles, as concluded in [25]. In addition, Šárpataky et al. summarized that the AC breakdown voltage has been enhanced from 4% up to 75% based on the type of insulating oil, the size of nanoparticles, and the nanoparticles’ concentrations [26]. Recently, Maher et al. measured the dielectric dissipation factor (DDF) after thermal and electrical faults on different nanofluids in power transformers. The results showed that the addition of nanoparticles to mineral oil decreased the DDF after the presence of thermal and electrical faults. Thus, the presence of nanoparticles (such as SiO_2_, Al_2_O_3_, and TiO_2_) inside the mineral oil increases its lifetime with better performance [27]. Abdali et al. showed that the insertion of 0.01% weight percentage of diamond nanoparticles inside mineral oil can reduce the hotspot temperature to 5.1 °C and enhance the coolant properties of the mineral oil [28]. Finally, Ghoneim et al. introduced a new approach to transformer tap changer maintenance using insulating oil-based nanoparticles [29].

From the abovementioned wide survey, most literature articles were conducted with nanofluids in power system apparatuses, especially power transformers. Contrary to that, the application of nanofluids with circuit breakers is very limited. Hence, in [30], oil-based nanofluids were used to treat the carbonization occurring at the contact points of a molded case circuit breaker, with a positive impact on the electrical properties of this circuit breaker. Other studies by Dessouky et al. used nanofluid as a practical application for the internal maintenance of miniature circuit breakers (MCB) and molded case circuit breakers (MCCB) as obvious in [31,32], respectively. However, no articles have investigated a potential real application of such nanofluids in power system apparatuses, namely circuit breakers. This study contains a new contribution to the industrial sector of circuit breaker manufacturer that considers a new idea in applying nanofluids inside circuit breakers and that simulates the temperature distribution and the generated heat energy during the making process of the minimum oil circuit breaker. Furthermore, to the author’s experience and knowledge, most researchers care about the application of nanofluids in power transformers and other electrical applications except circuit breakers. There is no one who simulates the thermal distribution and heat energy of nanofluids inside the minimum oil circuit breaker using a numerical thermal model with detailed parameters.

To tackle the aforementioned issue, this paper is considered the first study that tests the thermal performance of a real circuit breaker model filled with nanofluids. In addition, this article introduces the impact of different modified insulating oils by different types and concentrations of nanoparticles on the produced temperature and generated heat energy in the MOCB during its making process. The making process means the transition of contacts to convert the circuit breaker from the switch-off state to the switch-on position. MOCB is modeled by COMSOL Multiphysics software V.5.5. Temperature profile and generated heat energy in the insulating oils have been introduced at the different positions of the movable contact inside the arc quenching house. The insulating oil samples used in this simulation are mineral-oil-based nanofluids with different thermal conductivities as per the considered samples discussed in the next sections. We studied their effect on the evaluated parameters. The article’s contributions can be highlighted as follows:

- Proposing the use of nanofluids in MOCB as a real application by investigating nanofluids’ impacts on their thermal performance.
- Developing a thermal model for MOCB using the finite element method with more detailed parameters.
- Investigating the impact of nanoparticle’s types and concentrations on heat energy generation and temperature distribution with clarifying the corresponding physical mechanisms.

## 2. Considered Oil-Based Nanofluids

The enhancement of the thermal conductivity of insulating oils is highly required for the different insulating, cooling, and arc quenching applications. As mentioned in the previous section, the dispersion of certain types of nanoparticles into the base insulating oil with different weight percentages (wt%) can achieve this enhancement of oil thermal properties. In this paper, two types of insulating oils are selected (mineral oil and synthetic ester oil). Furthermore, two types of nanomaterials are selected to investigate the mitigation of thermal stress from the MOCB due to the presence of these nanomaterials in the insulating oils. These two nanomaterials are amorphous graphene nanosheets (N-GS) [16] and CaCu_3_Ti_4_O_12_ (CCTO) nanoparticles [21]. The selection of these nanomaterials is meant to achieve high thermal conductivity nanofluids, as introduced in [16,21].

**Figure 2:**
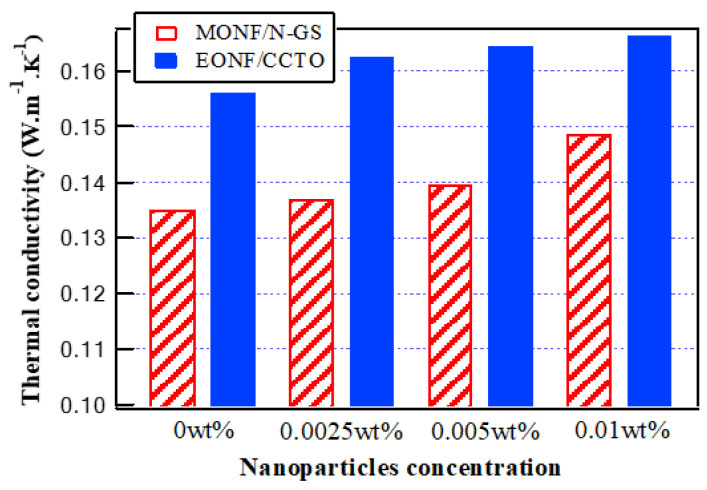
Thermal conductivity of oil-based nanofluids at various nanoparticle concentrations.

In [16], the dispersion of 0.0025, 0.005, and 0.01 wt% of amorphous graphene nanosheets into the base mineral oil enhanced its thermal conductivity by about 1.5%, 3.3%, and 10%, respectively. While in [21], the dispersion of 0.0025, 0.005, and 0.01 wt% of CCTO nanoparticles into the base synthetic ester oil enhanced its thermal conductivity by about 4.2%, 5.3%, and 6.5%, respectively. Figure 2 represents the thermal conductivities of the mineral oil nanofluid samples (MONF) and synthetic ester oil nanofluid samples (EONF), with standard unit (W·m^−1^·k^−1^), obtained from references [16,21].

## 3. Thermal Modelling of MOCB

In this section, a thermal model of a 66 kV MOCB is simulated using COMSOL Multiphysics software. The selected space dimension is 2D and the applied Multiphysics mode is the coupled interface between the electric currents and the heat transfer in fluids based on the finite element method. As introduced in the COMSOL Multiphysics software reference manual, the stationary electric currents model can be presented as follows [33]:

**J** = σ **E** + **J_e_** (1)

where **J** means the current density, σ denotes the electrical conductivity (S/m), **J_e_** is an externally produced current density (A/m^2^), and **E** means the electric field strength (V/m).

The continuity equation can be in the static form as:

∇**J** = –∇(σ ∇V − **J_e_**) = 0 (2)

where V is the electrical potential (V). The heating source due to the current source based on current density and electrical potential can be written as [33]:

Q _(j,V)_ = **J**·**E** (3)

The electric field strength can be expressed as:

**E** = −∇V (4)

Therefore,

Q = (σ ∇V − **J_e_**)·∇V (5)

In this model, the external current density, **J_e_** does not share in the losses because there is no electric field associated with it. Accordingly, its value is set at 0 A/m^2^ [34]. The dependent variable for the computation of this electric model is electrical potential (V), whose value at the fixed contact is 66/$\sqrt{3}$ kV and, at the movable contact, is grounded. The electric potential contact (fixed contact) implements an electric potential V_o_ as the boundary condition V = V_o_. The ground contact (movable contact) provides zero potential nodes as the boundary condition V = 0. The porcelain enclosure of the MOCB is modeled as electric insulation with boundary condition (**n**·**J** = 0) means that no electric current flows into this boundary. On the other hand, the physical model of heat transfer in the fluid can be summarized as heating source Q (w/m^3^) [34]:

ρ C_p_ ∂T/∂t + ρ C_p_ **u**·∇T − ∇·(k_c_ ∇T) = Q, (6)

where ρ depicts the fluid density (kg/m^3^), followed by C_p_ means the fluid heat capacity at constant pressure (J/(kg·K)), **u** represents the fluid velocity field (m/s), k_c_ means the fluid thermal conductivity (W/(m·K)), and the dependent variable in this model is the temperature T (K) that is initially set at room temperature of 300 K as a boundary condition. The fluid inside MOCB in the making process is considered stationary. Thus, the heat transfer in this model is dependent on the fluid heat conduction, not the fluid heat convection, where the fluid heat conduction depends on the fluid thermal conductivity, but the fluid heat convection depends on the fluid velocity that equals zero according to our assumption. The second item in the model assumption is that the thermal conductivity is fixed during the making process of MOCB due to the short time span of such a process.

In this study, the coupling between the electric currents model and the heat transfer in fluids deals with the heating source term of Q_e_ (W/m^3^) that is expressed in Equations (3) and (7). Since the making process instant is short, the oil’s thermal conductivity is considered a constant value at this instant.

ρ C_p_ ∂T/∂t − ∇·(k_c_ ∇T) = Q_e_ (7)

Equations (3) and (7) illustrate the parameters considered in the interfacing between the electric current and the heat transfer in this model. The heating source of the model depends upon the fluid parameters (ρ, C_p_, and k_c_) as shown in Equation (7), and upon the electric parameters (**J** and **E**), as shown in Equation (3).

**Figure 3:**
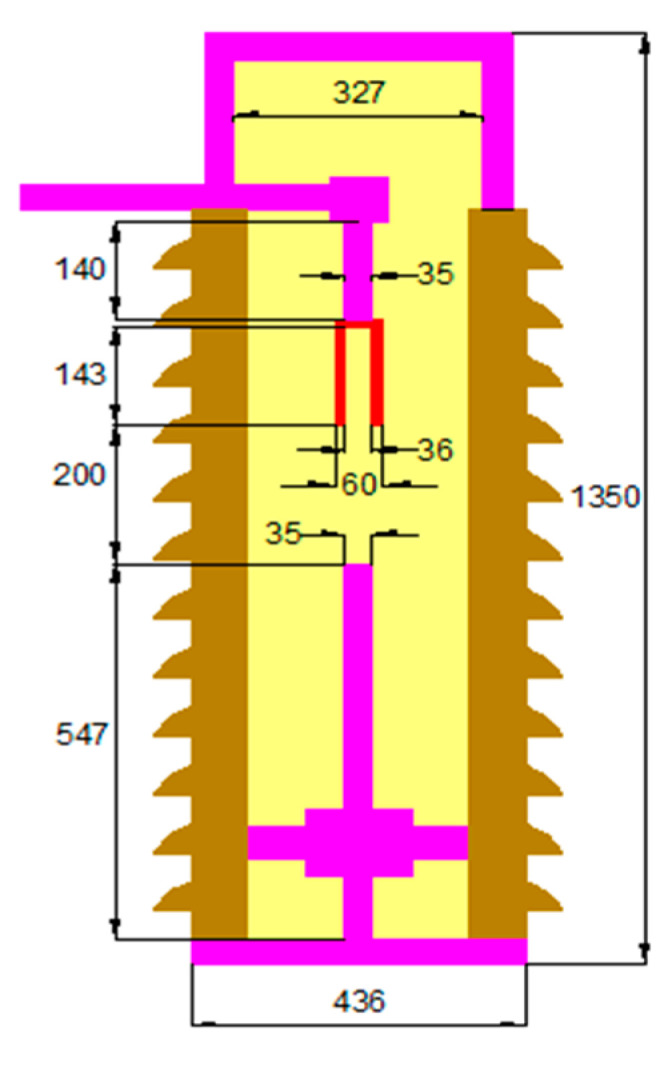
Internal view of the simulated MOCB with millimeters dimensions.

As shown in Figure 1, the internal construction of one pole of MOCB is presented. The high-voltage terminal is connected to the fixed contact, while the movable contact opens or closes the high-voltage terminal with the system through a mechanical operating mechanism. The model of a typical 66 kV MOCB is built using COMSOL Multiphysics software based on the dimensions shown in Figure 3. This model is built according to technical data (construction, dimensions, special parts, etc.) from relevant datasheets.

This simulation not only introduces the temperature distribution inside the minimum oil circuit breaker in the making process before arcing spread between its contacts but also evaluates its internal energy. The internal energy U (J/kg) represents the entire energy of an enclosed system that is related to the heat transfer between two different temperature objects. The following equation shows that the internal energy inside MOCB depends upon the oil heat capacity and the temperature difference between oil molecules.

U = C_p_·ΔT (8)

**Figure 4:**
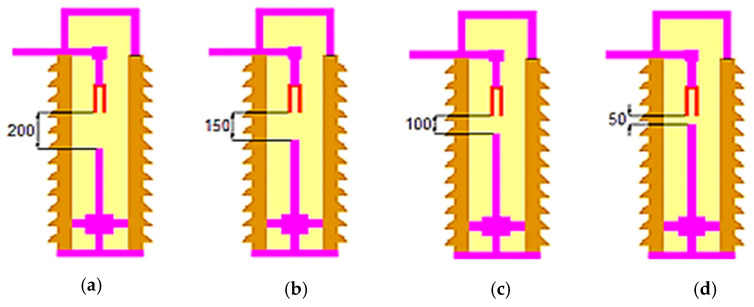
MOCB model at different positions of the movable contact inside the arc quenching house: (**a**) 200 mm; (**b**) 150 mm; (**c**) 100 mm; (**d**) 50 mm.

Moreover, the electric potential between the contacts and current density will be responsible for heat generation, as illustrated in Equation (3) above. In order to investigate this problem with a stationary COMSOL Multiphysics study, a certain distance between the arc quenching house and the movable contact was considered. The gap between the arc quenching house and the movable contact at the completely open state of the modeled circuit breaker is 200 mm. The simulation was created by a set of this distance at 75%, 50%, and 25% of the completely open distance (i.e., at 150, 100, and 50 mm, respectively) as shown in Figure 4. Hence, the limitation of the thermal model is that the MOCB thermal model is simulated in 2D space dimension and stationery mode, in addition to the consideration of a single-phase unit that is symmetrical for the simulation of the other two phases (means, 3-phase MOCB units). We use the phase voltage that is equal to the line voltage divided by $\sqrt{3}$. Finally, the heat transfer in this model is based on fluid heat conduction, not fluid heat convection.

**Figure 5:**
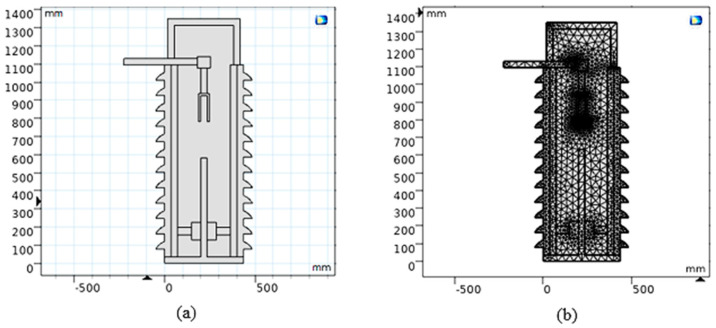
MOCB model after creation with 2D space dimension on the COMSOL software window: (**a**) before mesh building up; (**b**) after mesh building up.

Figure 5a presents the model of MOCB after creation with the 2D space dimension on the COMSOL Multiphysics software window. Figure 5b introduces the mesh presentation of extra fine finite element size to solve the model equations to evaluate the temperature distribution and internal heat energy inside MOCB. The gap between the arc quenching house and the movable contact presented in Figure 5 is 200 mm (i.e., completely open state). Note that the detailed thermal model parameters of MOCB and the datasheet parameters of various oil types have been extracted from real experimental data.

## 4. Results and Discussion

The simulation of a thermal model of one pole of a 66 kV MOCB is presented in this section using COMSOL Multiphysics. The simulation is introduced when the MOCB is filled by MONF/N-GS with different concentrations of N-GS. On the other hand, the simulation is repeated when the MOCB is filled by EONF/CCTO with different concentrations of CCTO. The simulation is processed for all nanofluid samples at different gap distances between the arc quenching house and the movable contact (150 mm, 100 mm, and 50 mm). The maximum temperature values inside the circuit breaker have been presented on the top of the color distribution bar beside each sub-figure.

### 4.1. A 150 mm Gap Distance

**Figure 6:**
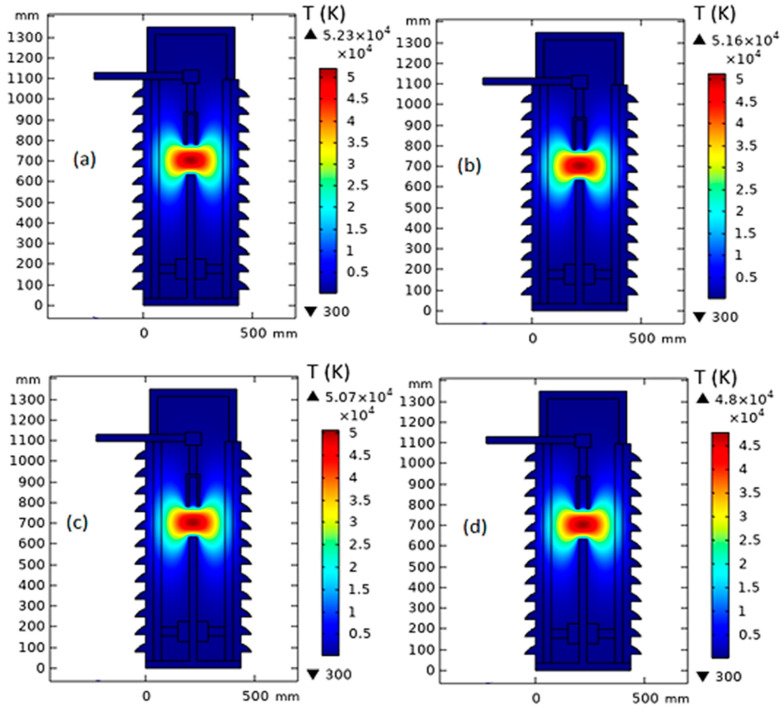
Temperature distribution inside MOCB in the case of MONF/N-GS at a gap distance of 150 mm with different nanoparticle concentrations: (**a**) 0 wt%, (**b**) 0.0025 wt%, (**c**) 0.005 wt%, and (**d**) 0.01 wt%.

**Figure 7:**
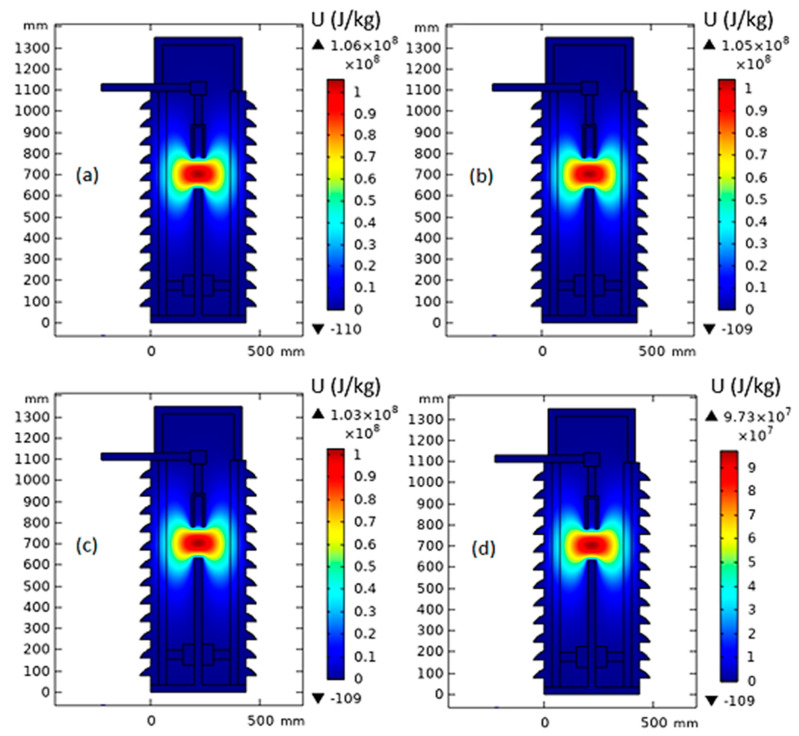
Internal heat energy inside MOCB in the case of MONF/N-GS at a gap distance of 150 mm with different nanoparticle concentrations: (**a**) 0 wt%, (**b**) 0.0025 wt%, (**c**) 0.005 wt%, and (**d**) 0.01 wt%.

Figure 6 shows the temperature distribution inside MOCB in the case of a 150 mm gap distance between the arc quenching house and the movable contact with the insulating medium of MONF/N-GS. From the obtained results, due to the increase in nanoparticle concentrations of 0.0025%, 0.005%, and 0.01%, the thermal stress inside the MOCB decreases from 52,300 K to 51,600 K, 50,700 k, and 48,000 K, respectively. Furthermore, as presented in Figure 7, the internal heat energy decreases from 106 × 10^3^ kJ/kg to 105 × 10^3^ kJ/kg, 103 × 10^3^ kJ/kg, and 97.3 × 10^3^ kJ/kg with the increase in nanoparticle concentration by 0.0025%, 0.005%, and 0.01%, respectively.

**Figure 8:**
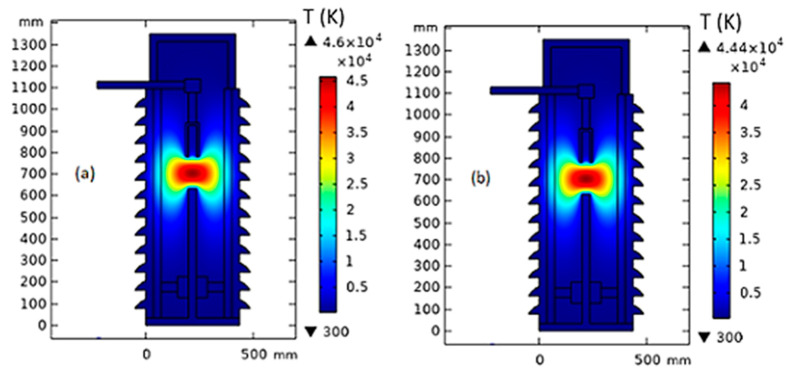

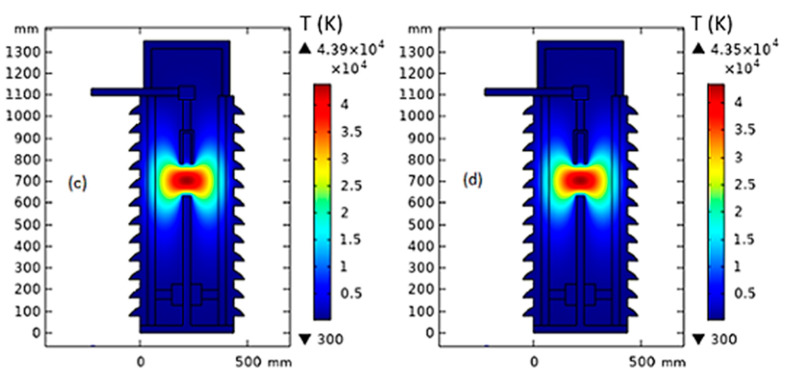
Temperature distribution inside MOCB in the case of MONF/CCTO at a gap distance of 150 mm with different nanoparticle concentrations: (**a**) 0 wt%, (**b**) 0.0025 wt%, (**c**) 0.005 wt%, and (**d**) 0.01 wt%.

**Figure 9:**
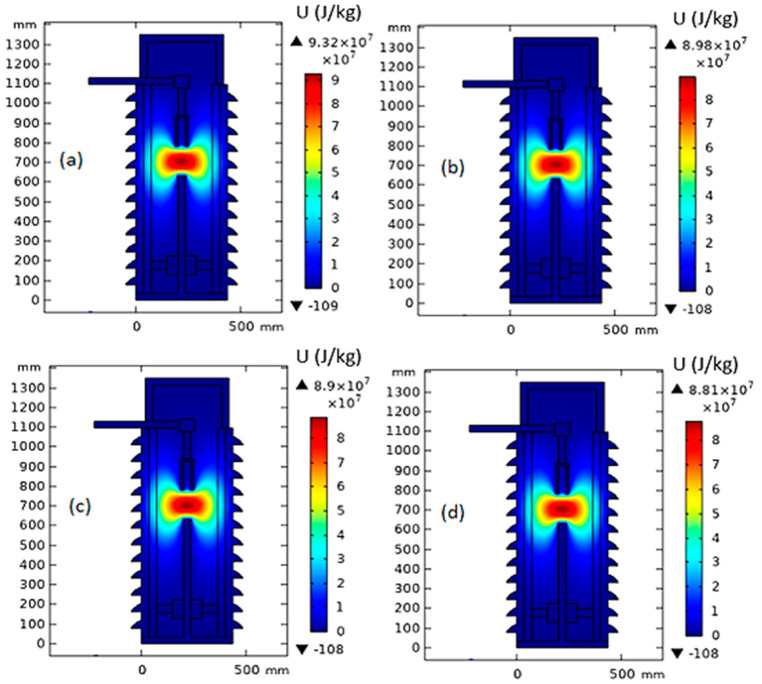
Internal heat energy inside MOCB in the case of MONF/CCTO at a gap distance of 150 mm with different nanoparticle concentrations: (**a**) 0 wt%, (**b**) 0.0025 wt%, (**c**) 0.005 wt%, and (**d**) 0.01 wt%.

On the other hand, in the case of EONF/CCTO, the generated temperature around the arc quenching house at 150 mm gap distance is decreased from 46,000 K to 44,400 K, 43,900 K, and 43,500 K, respectively, with the increase in CCTO loading changed by 0.0025%, 0.005%, and 0.01%, as presented in Figure 8. While the internal heat energy is decreased from 93.2 × 10^3^ kJ/kg to 89.8 × 10^3^ kJ/kg, 89 × 10^3^ kJ/kg, and 88.1 × 10^3^ kJ/kg, respectively, as shown in Figure 9.

**Figure 10:**
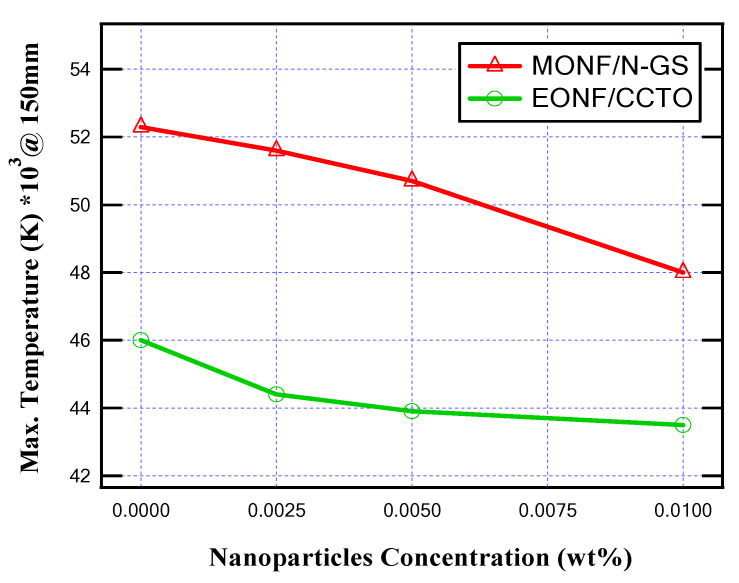
Maximum temperature for different nanoparticle concentrations at 150 mm gap distance.

**Figure 11:**
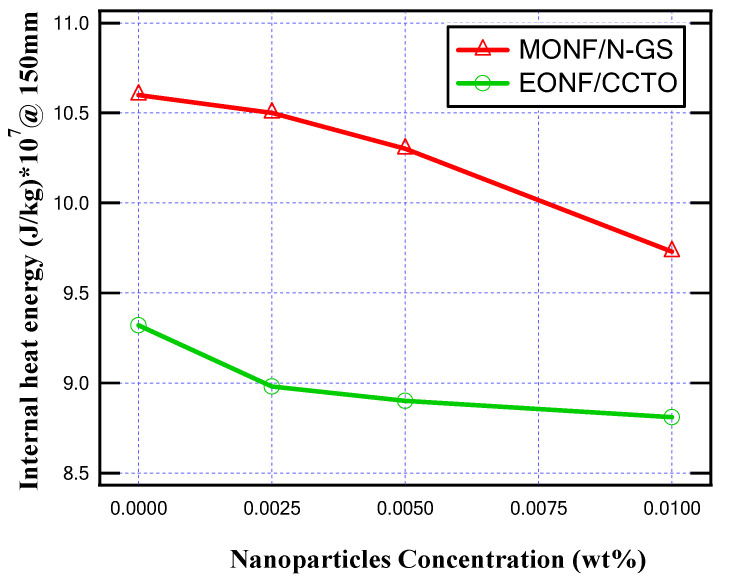
Maximum internal heat energy for different nanoparticle concentrations at 150 mm gap distance.

From the obtained results, as illustrated in Figure 10 and Figure 11, the produced temperature was reduced due to the better thermal conductivity of the insulating oil, and, also, the generated heat energy inside MOCB decreased. About a 8.3% and 4.7% decrease in the oil temperature was achieved at 0.01 wt% of N-GS and CCTO, respectively. On the other hand, the internal heat energy is reduced by 8.2% and 5.5% at 0.01 wt% of N-GS and CCTO, respectively.

### 4.2. A 100 mm Gap Distance

**Figure 12:**
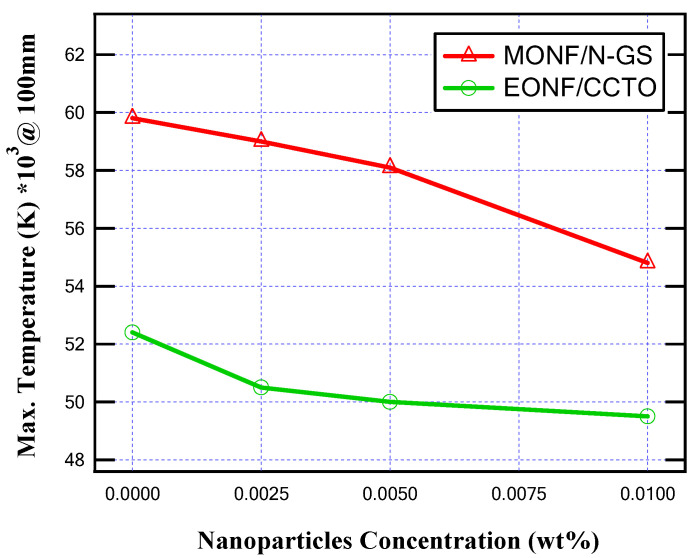
Maximum temperature for different nanoparticle concentrations at 100 mm gap distance.

**Figure 13:**
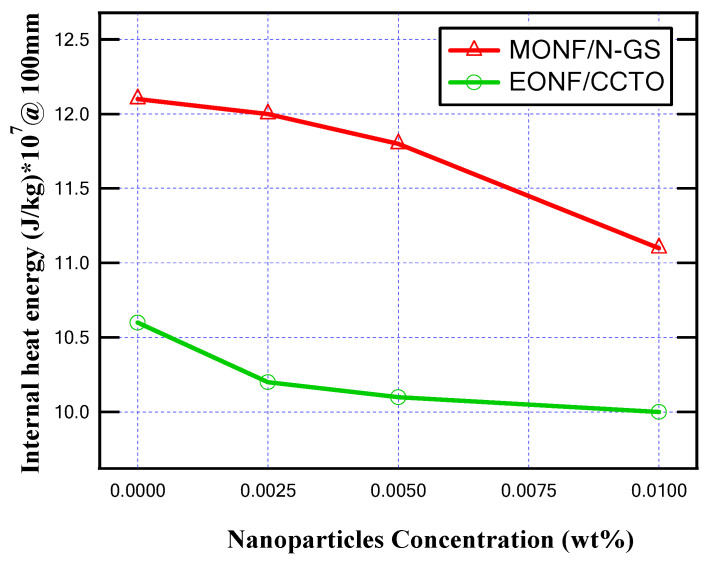
Maximum internal heat energy for different nanoparticle concentrations at 100 mm gap distance.

In this subsection, the simulation is introduced when the MOCB is filled by MONF/N-GS and repeated when it is filled by EONF/CCTO at a 100 mm gap distance with different concentrations of nano-additives. The maximum temperature values due to the making process of MOCB contacts at 100 mm separation for different insulating oils are presented in Figure 12. On the other hand, Figure 13 introduces the maximum internal heat energy for all insulating oil samples.

From the obtained results, as summarized in Table 1, the produced temperature is reduced due to the better thermal conductivity of the insulating oil. In the case of MONF, the larger the N-GS wt%, the lower the generated temperature inside the insulating oil. A percentage decrease of about 8.5% in the oil temperature is achieved at 0.01 wt% of N-GS compared with the base mineral insulating oil. On the other hand, In the case of EONF, about 5.5% mitigation in the oil temperature inside the MOCB is provided at 0.01 wt% of CCTO compared with the base ester insulating oil.

Regarding the internally generated heat energy, due to the increase in nanoparticles loading ratio, the generated heat energy decreased. In the case of MONF, the maximum generated heat energy is decreased at 0.01 wt% of N-GS by about 8.3% compared with the base mineral insulating oil. While, in the case of EONF, the maximum generated heat energy is decreased at 0.01 wt% of CCTO by about 5.7% compared with the base ester insulating oil. Comparing the worst sample (base mineral oil) and the best sample (EONF/CCTO at 0.01 wt%), it is found that a decrease in internal temperature and generated heat energy inside the MOCB was achieved by about 17%.

### 4.3. A 50 mm Gap Distance

**Figure 14:**
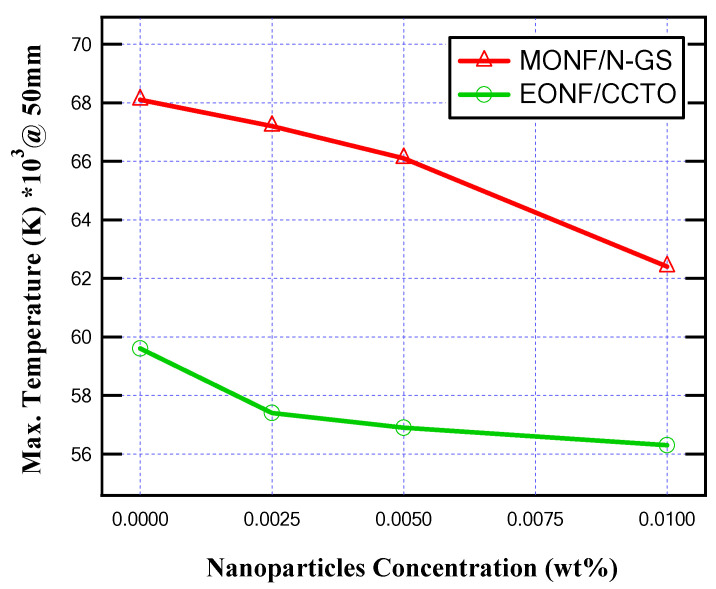
Maximum temperature for different nanoparticle concentrations at 50 mm gap distance.

**Figure 15:**
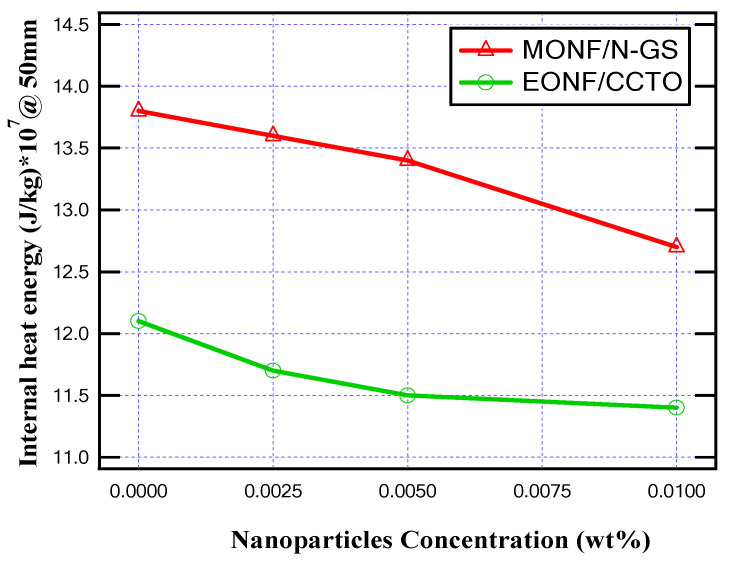
Maximum internal heat energy for different nanoparticle concentrations at a 50 mm gap distance.

As simulated in the cases of 150 mm and 100 mm gap distances between the arc quenching house and the movable contact, the simulation is repeated at a 50 mm gap distance. From the obtained results, as presented in Figure 14 and Figure 15, the maximum produced temperature and maximum generated heat energy inside MOCB are reduced due to the higher thermal conductivity of the insulating oil as obtained in the cases of 150 mm and 100 mm gap distances between the arc quenching house and the movable contact. In this case of gap distance, the maximum temperature and maximum internal heat are reduced by about 8.4% and 8%, respectively, for MONF/N-GS, and it was reduced by about 5.5% and 5.8%, respectively, for MONF/CCTO at the nanoparticle’s concentration (i.e., 0.01 wt%).

## 5. Physical Mechanisms

The MOCB contacts are completely immersed in the oil. This study aims to investigate the generated heat during pre-arcing in the making process. The heat is generated due to the effect of the applied electric field. Accordingly, the oil’s thermal conductivity is considered a constant value for a certain position of contacts. Around contacts, the high temperatures produced cause carbonization of the oil molecules to a certain level, then creates small carbon impurity particles and hydrogen gas that provide a media for arc spread. In some cases, the produced heat inside the MOCB during the making process can represent a more severe condition than that of the breaking condition, such as auto-reclosing on an existing fault.

As presented, all oil samples increased the maximum temperature and internal heat energy in a range of about 13.5% to 14.5%, with the variation of the gap distance from 150 mm to 100 mm and 100 mm to 50 mm. As shown in the obtained results, the smaller the gap distance between the arc quenching house and the movable contact, the higher the thermal stress on the MOCB. This case is presented at the gap distance of 50 mm when the insulating oil type is base mineral oil. The maximum produced temperature and maximum internal heat are 68,100 K and 138 × 10^3^ kJ/kg, respectively. This returns to the presence of a higher electric field on the insulating oil between contacts compared with 100 mm and 150 mm gap distances. The risk of this thermal stress is minimized with the existence of nanoparticles that improve the thermal properties of the insulating oil. Consequently, the mitigation of the produced temperature and the generated heat energy inside the MOCB returns to the high thermal conductivity of the different nanofluids. The higher the thermal conductivity, the better the coolant effect that decreases the maximum temperature and the internal energy while increasing the circuit breaker lifetime by keeping it with lower thermal stress. To further verify this conclusion, many evaluations of the maximum temperature and the internal energy at 100 mm gap separation have proceeded for many different insulating oils with different thermal conductivities quoted from references [35,36,37]. Table 2 presents the different oils considered in these studies and the corresponding computed maximum temperature and internal energy. When the thermal conductivity of insulating oil is increased by the presence of the nanomaterials, the temperature and internal energy will be decreased to provide higher cooling safety for the MOCB components.

These improvements in thermal conductivity return to many reasons. These reasons can be summarized as: (i) Interfacing between the solid nanoparticles and the insulating oil plays an effective role in the heat conduction process [38]. (ii) Brownian motion, i.e., the nanoparticles’ random anarchic motion, helps the phonons devolve from one nanoparticle to another. This motion provides a better heat convection process [39]. (iii) Cluster formation of nanoparticles leads to direct heat transfer from one molecule to another with low heat loss to improve the thermal conductivity of nanofluids [40]. Regarding the insulating oil type, it is seen that the usage of synthetic ester oil is more recommended than mineral oil. This recommendation returns to not only synthetic ester oil having better thermal conductivity than mineral oil, but also synthetic ester oil has many other advantages over mineral oil, including higher flash point, better lubrication, greater moisture tolerance, and superior oxygen stability [41].

**Figure 16:**
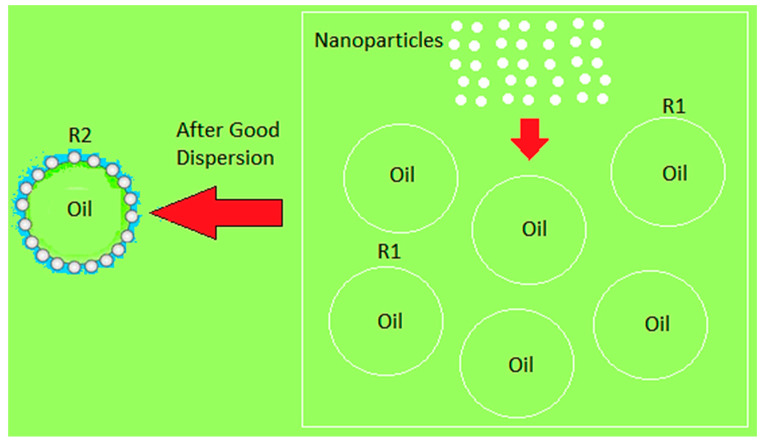
Dispersion and interface of nanoparticles with insulating oil molecules.

**Figure 17:**
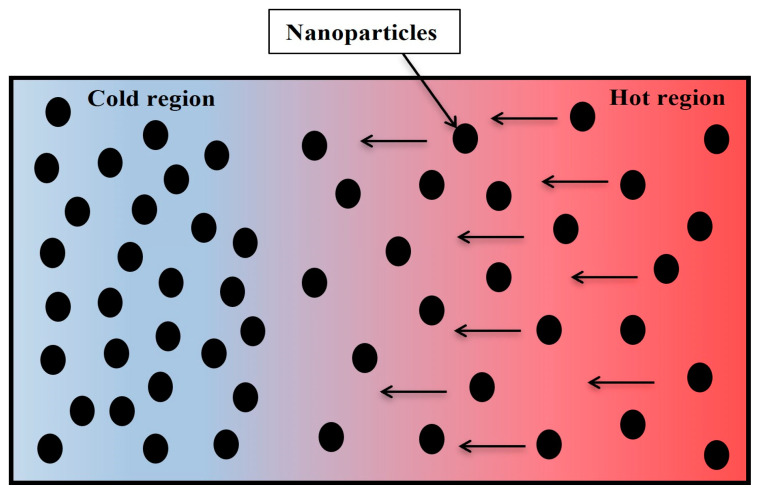
Thermophoresis procedure inside insulating oil-based nanoparticles.

For more clarification of the enhancement of mineral oil thermal conductivity that improves the temperature distribution and heat energy inside MOCB as simulated above, it shall be known that when certain types of nanoparticles are dispersed inside the insulating oil, the thermal conductivity of this oil is improved, due to numerous reasons. Figure 16 clarifies the interface between the nanoparticles and the oil molecules. Before the insertion of nanoparticles inside the insulating oil, the radius of oil molecules is assumed to be R1, and, after good dispersion, is R2. Due to the perfect interface, the interface charge depends on the distribution of nanoparticles’ mobility and the flux of charge carriers [42]. It is presented that the radius R2 is larger than R1, which produces a higher molecule surface for a better heat conduction process. This behavior explains the enhancement of the thermal characteristics of mineral oil as obtained in the results above. Moreover, this enhancement can be attenuated to the Brownian motion of nanoparticles within insulating oil subjected to a steady-state temperature gradient. This phenomenon is known as thermophoresis [43]. As a result of the thermophoresis effect, the nanoparticles drifted from the hot regions to the cold regions, dissipating the heat, and thereby enhancing the internal heat energy and temperature distribution inside MOCB, as presented in Figure 17. The thermophoresis procedure improves the temperature distribution and heat energy inside the oil as well. For example, as shown in Table 2, sample S9 has a lower thermal conductivity of 0.1204 W/(m·K) and achieved a maximum temperature of 66.5 × 10^3^ K and heat energy of 13.5 × 10^7^ J/kg. However, due to the higher thermophoresis that provides higher thermal conductivity with 0.255 W/(m·K) to the oil sample, S15 achieved a maximum temperature of 33.8 × 10^3^ K and heat energy of 6.84 × 10^7^ J/kg, i.e., the higher the insulating oil with a high thermophoresis, the higher thermal conductivity with lower thermal stresses on the oil molecules that provide better performance and superior lifetime for MOCB.

## 6. Conclusions

In this work, 66 kV MOCB is modeled by COMSOL Multiphysics software. A Multiphysics mode of the interface between the electrical potential and the heat transfer in fluids is used to evaluate the internal temperature and the internally generated heat energy inside the MOCB during the making process of its contacts at different positions of the movable contact. The results showed that the worst thermal stresses that appear on the MOCB are at a 50 mm gap distance between the arc quenching house and the movable contact. At this gap distance, in the case of MONF, with the increase in N-GS wt%, the internal temperature and the generated heat energy inside MOCB decreased by about 8.4% and 8%, respectively. While, in the case of EONF, with the increase in CCTO wt%, the internal temperature and the generated heat energy inside MOCB decreased by about 5.5% and 5.8%, respectively. The thermal stress on MOCB is minimized by 17.5% by comparing the base mineral oil and the sample of EONF/CCTO at 0.01 wt%. This improvement of thermal properties returns to the interface between the solid nanoparticles and the insulating oil, the random anarchic motion of the nanoparticles, and their cluster formation.

## Figures and Tables

**Table 1:** Maximum temperature and maximum internal heat energy of MONF/N-GS samples and EONF/CCTO samples at 100 mm gap distance.

Nanoparticles Concentration (wt%)		0	0.0025	0.005	0.01
Maximum Temperature (K) × 10^3^	MONF/N-GS	59.8	59	58.1	54.8
	EONF/CCTO	52.4	50.5	50	49.5
Maximum internal energy (J/kg) × 10^7^	MONF/N-GS	12.1	12	11.8	11.1
	EONF/CCTO	10.6	10.2	10. 1	10

**Table 2:** Maximum temperature and internal energy for many different Nano-oils.

Sample ID	Oil Type	Nano Type	wt%	k_c_	T × 10^3^	U × 10^7^
S1	MO	N-GS	0.0025	0.137	59.0	12.0
S2	MO	N-GS	0.005	0.1395	58.1	11.8
S3	MO	N-GS	0.01	0.1486	54.8	11.1
S4	EO	CCTO	0.0025	0.1625	50.5	10.2
S5	EO	CCTO	0.005	0.1643	50.0	10.1
S6	EO	CCTO	0.01	0.1662	49.5	10.0
S7	MO	BN	0.05	0.12075	66.3	13.5
S8	MO	BN	0.1	0.1212	66.1	13.4
S9	MO	Fe_3_O_4_	0.05	0.1204	66.5	13.5
S10	MO	Fe_3_O_4_	0.1	0.1205	66.5	13.5
S11	EO	TiO_2_	0.005	0.164	50.1	10.2
S12	EO	TiO_2_	0.01	0.166	49.5	10.0
S13	EO	TiO_2_	0.05	0.169	48.8	9.88
S14	EO	graphene	0.002	0.225	37.8	7.64
S15	EO	graphene	0.004	0.255	33.8	6.84
S16	MO	Al_2_O_3_	0.5	0.142	57.1	11.6
S17	MO	Al_2_O_3_	1	0.145	56.0	11.4
S18	MO	Al_2_O_3_	2	0.151	54.0	11.0
S19	MO	Al_2_O_3_	4	0.162	50.7	10.3
S20	EO	TiO_2_	0.005	0.164	50.1	10.2
S21	EO	TiO_2_	0.01	0.166	49.5	10.0
S22	EO	TiO_2_	0.05	0.169	48.8	9.88

## Data Availability

The data presented in this study are available on request from the corresponding author.

## Nomenclatures

MOCB	Minimum oil circuit breaker
SF_6_	Sulfur hexafluoride
Al_2_O_3_	Aluminum oxide (alumina)
CCTO	Calcium copper titanium oxide (CaCu_3_Ti_4_O_12_)
ZnO	Zinc oxide
DDF	Dielectric dissipation factor
SiO_2_	Silicon dioxide
TiO_2_	Titanium dioxide
MCB	Miniature circuit breaker
MCCB	Molded case circuit breaker
wt%	Weight percentages
N-GS	Amorphous graphene nanosheets
MONF	Mineral oil nanofluid samples
EONF	Synthetic ester oil nanofluid samples
MO	Mineral oil
EO	Ester oil
Fe_3_O_4_	Iron oxide
R1	Radius of oil molecules
R2	Radius of oil molecules after good dispersion
J	Current density
σ	Electrical conductivity
J_e_	Externally generated current density
E	Electric field strength
V	Electrical potential
V_o_	Boundary electrical potential of the fixed contact
Q	Heating source
ρ	Fluid density
C_p_	Fluid heat capacity at constant pressure
k_c_	Fluid thermal conductivity
u	Fluid velocity field
T	Dependent variable temperature in the model
Q_e_	Heating source term
ΔT	Temperature difference between oil molecules
U	Internal energy inside MOCB
